# Protein Fractions from Korean Mistletoe (*Viscum Album coloratum*) Extract Induce Insulin Secretion from Pancreatic Beta Cells

**DOI:** 10.1155/2014/703624

**Published:** 2014-05-14

**Authors:** Ki-Wook Kim, Seung-Hoon Yang, Jong-Bae Kim

**Affiliations:** ^1^School of Life Sciences, Handong Global University, Pohang, Gyungbuk 791-708, Republic of Korea; ^2^Department of Microbial Pathogenesis, Boyer Center for Molecular Medicine, Yale University School of Medicine, New Haven, CT 06510, USA; ^3^Department of Biological Chemistry, The Weizmann Institute of Science, 76100 Rehovot, Israel

## Abstract

Mistletoe (*Viscum Album coloratum*) has been known as a medicinal plant in European and Asian countries. Recent data show that biological activity of mistletoe alleviates hypertension, heart disease, renal failure, and cancer development. In this study, we report the antidiabetic effect of Korean mistletoe extract (KME). KME treatments enhanced the insulin secretion from the pancreatic **β**-cell without any effects of cytotoxicity. PDX-1 and beta2/neuroD known as transcription factors that regulate the expression of insulin gene were upregulated by treatment of the KME protein fractions isolated by ion-exchange chromatography after ammonium sulfate precipitation. Furthermore, these KME protein fractions significantly lowered the blood glucose level and the volume of drinking water in alloxan induced hyperglycemic mice. Taken together with the findings, it provides new insight that KME might be served as a useful source for the development of medicinal reagent to reduce blood glucose level of type I diabetic patients.

## 1. Introduction

Insulin is an essential hormone that maintains the blood and urinary glucose level. At transcriptional level, insulin gene expression is controlled by the specific transcription factors: pancreatic and duodenal homeobox factor-1 (PDX-1) and beta2/neuroD [1–4]. However, abnormal immune response destroys pancreatic *β* cell and impairs insulin secretion, leading to insulin dependent type I diabetes [5, 6]. It leads to severe complications such as vascular disease, renal failure, and heart disease [7–9]. Although exogenous insulin therapy through the insulin pump or subcutaneous insulin injection temporally delays the onset of complications, it finally results in type I diabetes mellitus [10–13].

Mistletoe (*Viscum album*) is a semiparasitic plant which grows on deciduous trees in Asian and European countries. It has been used as a traditional therapeutic reagent to lower blood pressure and modulate immune responses [14–18]. Previously, we reported that Korean mistletoe has a variety of immunological activities such as cytokine induction, NK cell activation, and adjuvant activity [19–21]. Specifically, lectins isolated from KME had antitumor activity to suppress tumor growth and to inhibit tumor-induced angiogenesis or metastasis [22–24]. Although it has been reported that KME and its lectins played critical role for immunological activity and antitumor activity [25], little is known about the antidiabetic effect of Korean mistletoe.

In this study, we revealed KME protein fractions without cellular damage induced to release insulin from the pancreatic *β* cell by activating transcription factors such as PDX-1 and beta2/neuroD required for insulin gene expression. Possibly, due to the elevation of insulin release by KME protein fraction, the blood glucose level and drinking water volume were significantly reduced in alloxan induced hyperglycemic mice [26, 27].

## 2. Materials and Methods

### 2.1. Reagents

RPMI 1640 without glucose, alloxan, and Krebs-Ringer bicarbonate (KRB) buffer were purchased from Sigma. Anti-PDX-1 and antineuroD, all from Santa Cruz Biotechnology, were used for immunoblotting. Rat insulin ELISA Kit (SHIBAYAGI) was used for insulin secretion. SYBR green master mix (Applied Biosystems) and RNA Easy-spin (iNtRON) were used for real-time PCR.

### 2.2. Cell Culture and Treatment

Rat pancreatic *β* cells, RINm5F cells, were provided by Pohang University of Science and Technology (POSTECH). RINm5F cells were cultured in a monolayer in RPMI 1640 medium (Gibco) supplemented with 10% fetal bovine serum (FBS, CAMBREX), 1% penicillin/streptomycin (CAMBREX), and 5.5 mM glucose at 37°C under a humidified 95%–5% (v/v) mixture of air and CO_2_. RINm5F cells were cultured in complete medium with 10% FBS to ~90% cell confluence and were then incubated with 0.5% FBS medium for 12~16 hours. After that, RINm5F cells were treated with KM extract. The supernatant was used for detection of insulin secretion and the attached cells were used for immunoblotting.

### 2.3. Animals

Specific pathogen-free 7-week-old imprinting control region (ICR) mouse strains were purchased from the Dae-Han Laboratory Animal Center, Republic of Korea. Mice were maintained in the Laboratory of Animal Experiment, Institute of Bioscience, Han-Dong Global University, under laminar air-flow conditions. Water and diets were supplied* ad libitum*. Each group was composed of 5 mice for checking blood glucose level and drinking water volumes.

### 2.4. Extraction and Purification of Korean Mistletoe

Mistletoe growing on oak (*Quercus variabilis *Blume) between December and February was harvested from Kangwon-do, Korea, and stored at −80°C until use. The nonlectin fractions (NLF) were isolated from an extract of Korean mistletoe plants using hydrolyzed *α*-lactose-Sepharose (Sigma) column chromatography after ammonium sulfate precipitation as described previously [24, 25]. Briefly, the extracts were dissolved in distilled water and filtered through a 0.45 *μ*m membrane (Whatmann). Then, they adjusted to 70% saturation with (NH_4_)SO_4_. The precipitate was resuspended in a small volume of phosphate-buffered saline (PBS), dialyzed against the same buffer, and applied to an *α*-lactose-Sepharose column which had been hydrolyzed for 2.5 hours. To remove the Korean mistletoe lectin (KML-C), the adsorbed material was eluted with a lactose-containing buffer (0.1 M lactose, pH 7.3). Unbounded proteins which were free of KML-C were pooled, dialyzed against distilled water, and freeze-dried. Each process was performed at 4°C. NLF dissolved in distilled water was partitioned by chloroform. Chloroformic extracts dissolved in phosphate buffer (PBS, 10 mM phosphate buffer containing 0.85% NaCl, pH 7.4) were applied to the DEAE-Sepharose Fast flow column (Amersham Pharmacia Biotech.) which had previously equilibrated with the same buffer. After column was washed with phosphate buffer again, remaining bound proteins were eluted with 0.5 M, 1 M, and 2 M NaCl (DEAE 0.5 M, 1 M, and 2 M fractions). DEAE fractions dissolved in PBS were applied to the Sep-Pak column (Waters Corporation) which had been previously equilibrated with the same buffer. The column was washed with PBS, and bound proteins were eluted with 20% ethanol (Sep-Pak 20%, SP20 fractions) and 60% ethanol (Sep-Pak 60%, SP60 fractions). Each eluted protein fraction was dialyzed against distilled water every 4 hours for 3 days and stored in −20°C until use.

### 2.5. FACS Analysis for Cytotoxicity

Cytotoxicity was analyzed by the annexin V and propidium iodide (PI, Merck) using flow-cytometry (FACS) machine (Beckman Coulter Epic XL). RINm5F cells (2 × 10^6^ cells) were seeded into 6-well plates and incubated for 12 hours. After washing, KM extract was treated with the cells and incubated in the 37°C with 5% CO_2_ for 30 minutes. Cells were carefully detached from the plates, and then detached cells were stained with the apoptosis detection kit containing annexin V-FITC and PI at ice in the dark condition. Stained cells were washed with FACS solution and living or dead cells were measured by FACS machine.

### 2.6. Measurement of Insulin

1 mL of RINm5F cells (4 × 10^5^ cells/well) was seeded into the 24-well plates (Falcon) and cultured in RPMI-1640 containing 11.1 mM glucose (Sigma). After incubation for 12 hr to allow attachment, cells were washed with Krebs-Ringer bicarbonate buffer (KRB) in 3 times. Then, cells were preincubated with 1 mL of KRB buffer for 30 minutes at 37°C. Cells were then incubated for 30 minutes with KM extracts. The supernatants were collected from each well and stored at −20°C until use for insulin secretion assay. An assay for the measurement of insulin secretion from RINm5F cells was carried out by rat insulin ELISA Kit (SHIBAYAGI) and detected by ELISA reader at 450 nm.

### 2.7. Real-Time PCR

Total RNA were extracted using easy-spin RNA extraction kit (Intron, Korea). cDNA were produced with 5 *μ*g of total RNA using Superscript (Invitrogen). In a florescent temperature cycler (LightCycler; Roche Diagnostics Ltd., Lewes, UK), 10 percent of each RT reaction was amplified in a 20 *μ*L PCR containing 4 mM MgCl_2_, 4 pmol of each primer, and 1x SYBR green master mix. Samples were incubated in the LightCycler for an initial denaturation at 94°C for 30 seconds, followed by 30 PCR cycles. Each cycle consisted of 95°C for 15 seconds, 55°C for 32 seconds, and 72°C for 32 seconds. 

*The Oligonucleotide Primers*

 For insulin are
   forward: 5′-ATA GAC CAT CAG CAA GCA GG-3′,
   reverse: 5′-CTC CAG TTG TGG CAC TTG CG-3′;
 For PDX-1 are
   forward: 5′-TAC GCG GCC ACA CAG CTC TAC AAG GAC-3′,
   reverse: 5′-CCA CTT CAT GCG ACG GTT TTG GAA CCA GA-3′;
 And  for *β*-actin are
   forward: 5′-AGG ATG CAG AAG GAG ATC ACT G-3′,
   reverse: 5′-GGG TGT AAC GCA ACT AAG TCA TAG-3′.

*β*-actin was used as an endogenous internal control. To confirm amplification of specific transcripts, melting curve profiles (cooling the sample to 65°C for 15 seconds and heating slowly to 95°C with continuous measurement of fluorescence) were produced at the end of each PCR procedure.

### 2.8. Western Blot Analysis

For Western blotting analysis, cells were lysed by resuspension in buffer A (25 mM Tris-HCl, pH 7.5, 2 mM Na orthovanadate, 0.5 mM EDTA, 10 mM NaF, 10 mM Na pyrophosphate, 80 mM *β*-glycerophosphate, 25 mM NaCl, 1% (v/v) Nonidet P-40) containing 1x complete protease inhibitor mixture (Roche Diagnostics) and then boiled for 10 min. After sonication, they were centrifuged for 15 min in 13,000 ×g. Protein concentration in the extract was determined using the BCA kit (Pierce). Protein samples were separated on SDS-PAGE and blotted onto nitrocellulose membrane (Amersham Biosciences). Blots were developed with the ECL kit (Pierce).

### 2.9. Generation and Analysis of Hyperglycemic Mice

ICR mice were grouped into several groups of five mice. Alloxan (70 mg/kg) was injected into mice via tail vein. Next day of alloxan injection, 200 *μ*L of DEAE eluted protein fractions from KM extracts (50, 100, 200, and 400 *μ*g/mL) was injected intraperitoneally. After 10 days, the same volumes of DEAE eluted protein fraction were injected intraperitoneally again. Blood was obtained from the tail vein of injected mice through heparinized capillary tube. Blood glucose levels were measured by the glucose measurement kit (Assan Pharmacia). Drinking water volumes were measured by a decimeter.

## 3. Results

### 3.1. KME Has No Cytotoxicity to Rat Pancreatic *β* Cell

**Figure 1:**
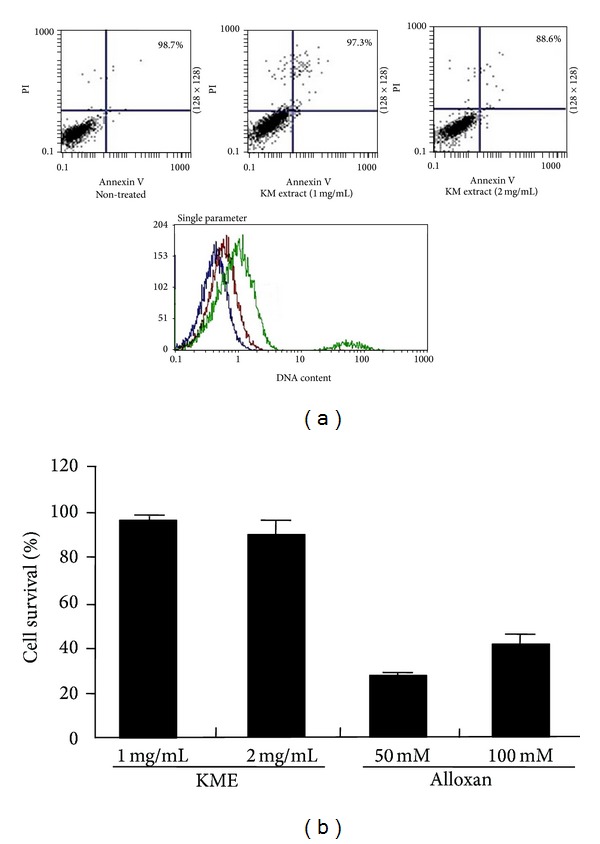
Cytotoxicity of pancreatic *β* cells by KME treatment. (a) Representative FACS analysis for PI and annexin V staining of RINm5F cell following KME treatment. Nontreated RINm5F cells (left), KME (1 mg/mL) treated RINm5F cell (middle), and KME (2 mg/mL) treated RINm5F cells (right). DNA contents of RINm5F cells under the KME treatment. Blue: nontreated, red: KME (1 mg/mL), and green: KME (2 mg/mL). (b) The cytotoxic activity of KME- or alloxan-treated cells was measured by XTT assay.

Due to the cytotoxicity of lectin A subunit contained in KME, we examined whether KME has the cytotoxic activity on rat pancreatic *β* cells (RINm5F). Apoptotic cells and necrotic cell were measured by annexin V and PI staining. As shown in Figure 1(a), most cells were alive after aqueous KM extract (1 mg/mL, 2 mg/mL) treatment. It indicates that RINm5 cell viability was not affected by KME treatment. To evaluate the cell proliferation as well as cytotoxicity, we incubated RINm5 cells with KME (1 mg/mL, 2 mg/mL) for 48 hours. The survival rate of RINm5 cell was measured by XTT assay. Well known as a chemical compound to specifically destroy pancreatic beta cells, alloxan severely damaged the RINm5 cells. However, RINm5 cells were not affected by KME at all (Figure 1(b)). These findings imply that KME is not toxic to the rat pancreatic *β* cells.

### 3.2. Lectin-Free KME Protein Fractions Induce Insulin Secretion on Rat Pancreatic *β* Cell

**Figure 2:**
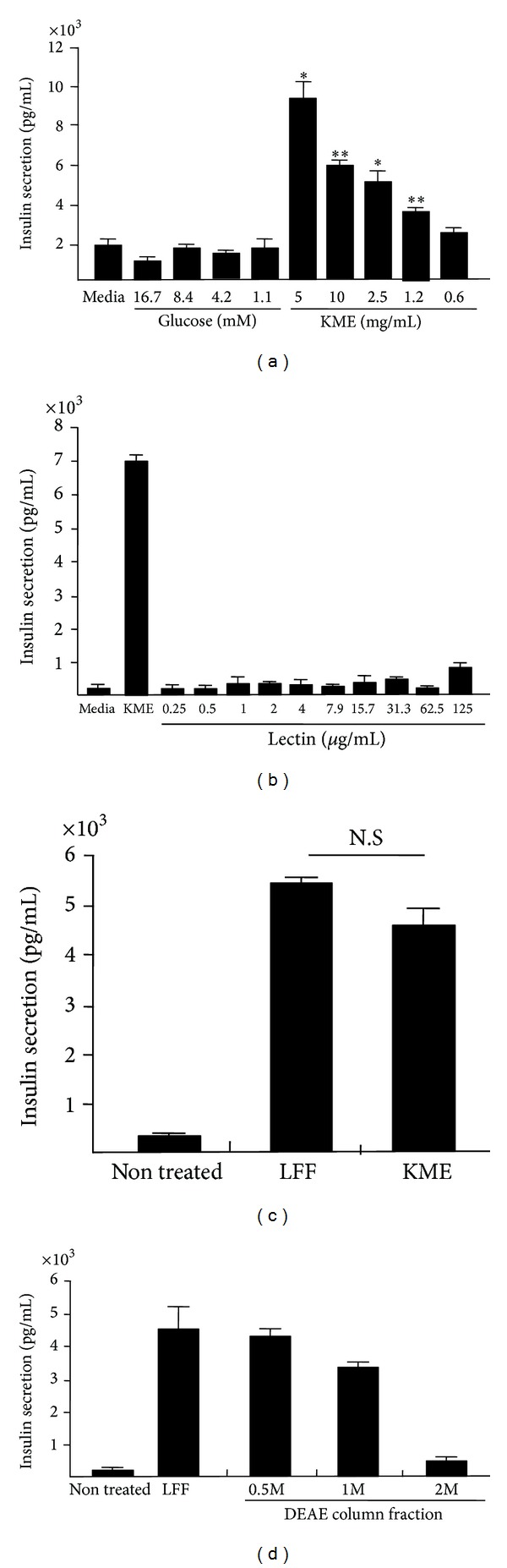
Insulin secretion of RINm5F cells by a variety of KME protein fractions. (a) Insulin secretion of RINm5F cell by KME dose dependent manner. (b) Insulin secretion of RINm5F cells by lectin (KML-C) isolated from Korean mistletoe. (c) Insulin secretion of RINm5F cells by the lectin-free KME and total KME. (d) Insulin secretion of RINm5F cells by protein fractions from KME isolated by ion-exchange chromatography.

To examine the potential to secrete insulin by KME, the insulin released from RINm5F cells was measured by ELISA after treatment of a several doses of KME. While glucose concentration had no effect to secrete insulin, KME had stimulatory effect on insulin secretion in a dose dependent manner (Figure 2(a)). Next, we investigated whether the lectin (KML-C) isolated from KME has potential to secrete insulin on RINm5F cells. Although the lectin (KML-C) isolated by KME was treated with the RINm5 cells in a dose dependent manner (0.25~125 *μ*g/mL) for 12 hours, insulin secretion was not detected in RINm5F cells (Figure 2(b)). However, lectin-free KME protein fraction (LFF) induced insulin secretion as similar as the KME treatments (Figure 2(c)). Thereafter, LFF was isolated from DEAE-Sepharose Column and finally divided into three fractions by concentration of 0.5 M, 1 M, and 2 M NaCl elution buffer, respectively. Both 0.5 M and 1 M NaCl eluted DEAE-Sepharose column fractions induced insulin secretion from RINm5F cells whereas 2 M NaCl eluted DEAE-Sepharose column fractions did not have insulin secretion ability (Figure 2(d)). Therefore, it suggested that purified protein factions from KME have an ability of insulin induction in rat pancreatic *β* cells.

### 3.3. Protein Fractions from KME Induce Insulin and PDX-1 Gene Expression

**Figure 3:**
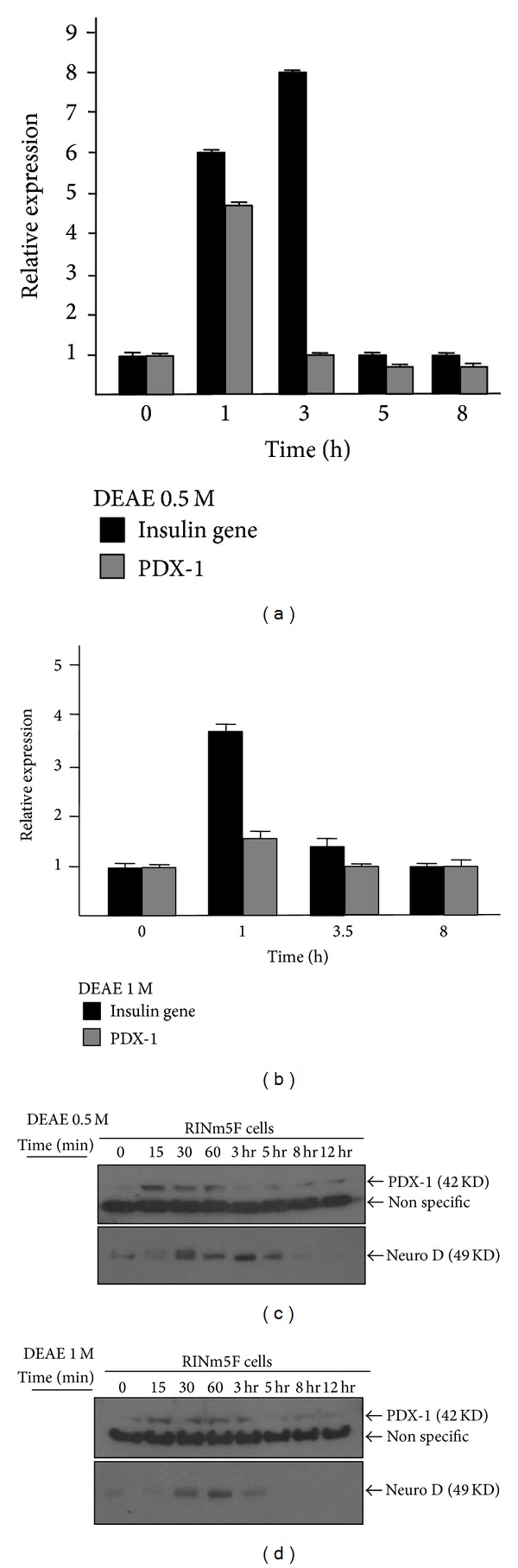
Insulin gene and transcription factor expression by DEAE-Sepharose fractionated KME protein fractions. ((a)-(b)) Time dependent mRNA expressions of insulin gene and PDX-1 gene in RINm5F cells under the treatment of (a) 0.5 M DEAE protein fractions and (b) 1 M DEAE. ((c)-(d)) Time dependent PDX-1 and beta2/neuroD protein expressions of RINm5F cells under the treatment of (d) 0.5 M DEAE protein fraction and (d) 1 M DEAE protein fraction.

The molecular mechanism of insulin secretion by protein fraction of KME remains unknown. To determine whether protein fractions of KME activate insulin gene in transcriptional levels, we analyzed the expression level of insulin gene and its related transcription factor, PDX-1, through the real-time PCR. After the treatment of 0.5 M DEAE protein fractions, insulin genes were peaked at three hours and PDX-1 genes were upregulated in an hour (Figure 3(a)). Although insulin genes and PDX-1 genes in 1 M-DEAE protein fraction treated RINm5F cells were relatively shown in low expression, they were also upregulated in an hour (Figure 3(b)).

Although the expression level of PDX-1 genes was not altered within 60 min, the expression level of insulin genes was significantly increased in real-time PCR analysis. To address this discrepancy, we determined to analyze the PDX-1 and beta2/neuroD expression in time dependent manner. Western analysis of PDX-1 expression revealed that this transcription factor was highly increased after 15 min of 0.5 M or 1 M DEAE protein fraction of KME treatment and its expression was gradually decreased. Similar to the PDX-1, beta2/neuroD is highly expressed within 60 min by the 0.5 M or 1 M DEAE protein fraction of KME (Figures 3(c) and 3(d)). These results strongly suggested that protein fractions of KME activate PDX-1 and beta2/neuroD transcription factors in early time point, eventually resulting in the activation of insulin gene.

### 3.4. Protein Fractions from KME Rescued the Insulin Secretion and Blood Glucose Level in Alloxan Induced Hyperglycemic Mice

**Figure 4:**
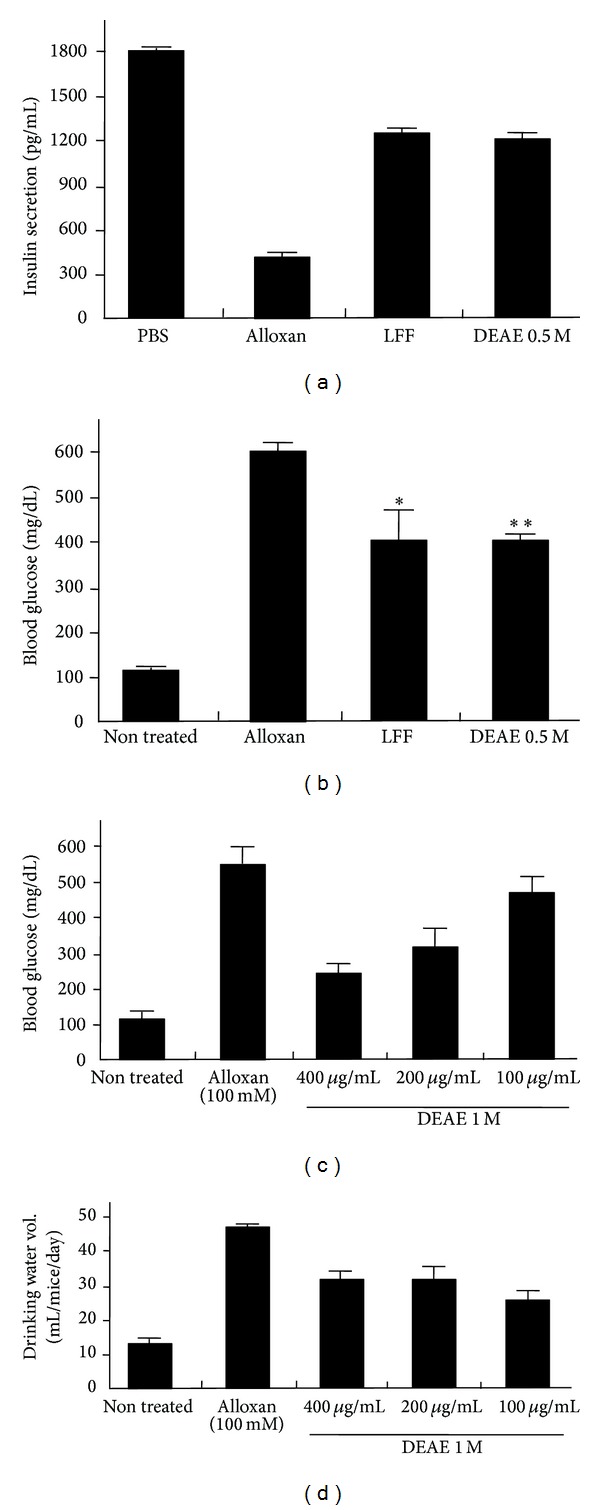
Antidiabetic effect of KME protein fractions on alloxan induced hyperglycemic mice. ((a)-(b)) Restoration of (a) insulin level and (b) blood glucose level of alloxan induced hyperglycemic mice by the lectin-free KME and 0.5 M DEAE protein fraction of KME. (c) Restoration of blood glucose level of alloxan induced mice by dose dependent 1 M DEAE protein fractions. (d) Reduction of drinking-water consumption of alloxan induced mice by 1 M DEAE protein fractions.

Next, we examined whether insulin secretion and blood glucose level could be rescued by introduction of the protein fractions of KME in vivo model. To this end, lectin-free fraction of KME or its 0.5 M DEAE protein fraction was injected into alloxan induced diabetic mice, respectively. Insulin secretion and serum glucose level were measured after 48 hours of KME fractionated protein injection. We found that insulin secretion was severely impaired by alloxan injection but it was partially restored by injection of the KME protein fractions (Figure 4(a)). Furthermore, blood glucose level was restored by 0.5 M DEAE protein fraction treated mice, compared to alloxan treated mice (Figure 4(b)). Also, it was confirmed that blood glucose level of alloxan induced diabetic mice was gradually reduced by dose dependent manner of 1 M DEAE protein fraction treatment (Figure 4(c)).

Similar to diabetic patients, alloxan induced diabetic mice consumed much more water than normal mice. To investigate whether the consumption of drinking water could be reduced by treatment of DEAE protein fractions of KME, we measured the volume of drinking water of diabetic mice in the presence or absence of DEAE protein fractions of KME. As expected, drinking water volume was slightly decreased regardless of the dose of DEAE protein fractions (Figure 4(d)). Collectively, these findings suggested that DEAE-Sepharose fractionated proteins from KM extract have an effective antidiabetic activity in alloxan induced hyperglycemia mice.

## 4. Discussion

It has been well known that Korean mistletoe has strong immunomodulatory effects such as immune cells activation, cytokines induction, and antitumor effects [19, 20, 22–24]. Despite the use of this plant as an effective traditional treatment for diabetes, little is known about the molecular mechanism for the antidiabetic effect of this plant [28].

In this study, we demonstrated the antidiabetic effect of the various KME protein fractions in vitro and in vivo molecular mechanisms of insulin secretion. First, we examined the induction ability of insulin secretion from pancreatic *β* cells by various protein fractions of KME through the activation of transcription factors such as PDX-1 and beta2/neuroD which regulate the insulin genes expression. Second, we found that pancreatic *β* cells were not impaired by KME treatment whereas these cells were severely destroyed by alloxan treatment. Our data support that KME directly activates the insulin genes and transcription factors associated with the insulin gene expression for the first time.

Initially, it has been expected that lectin of Korean mistletoe (KML-C) could have an effect on diabetes because of its diverse biological activities. However, the data showed in this study that KML-C is not related to the induction of insulin and lectin-free KME protein fractions (LFF) stimulate the secretion of insulin. Furthermore, we investigated the fact that protein fractions from KME separated by several purification methods induce insulin secretion from rat pancreatic *β* cells. Molecular signaling of insulin induction was controlled by activation of several transcription factors. Here, we demonstrated that PDX-1 and beta2/neuroD, which play a critical role in induction of insulin genes expression, were upregulated in KME treated pancreatic *β*-cells. Consistent with in vitro data, KME treatment restored the insulin secretion in alloxan induced hyperglycemic mice. Moreover, blood glucose level and drinking water volume were restored by KME treatment in alloxan induced hyperglycemic mice. These in vivo data support that KME is able to rescue destroyed pancreatic *β* cells or delay the pancreatic *β* cells destruction. As reported in Lyu et al., the lectin component of KME is able to promote cell proliferation [29]. Accordingly, we speculate that some specific components of KME regulate the proliferation or regeneration of pancreatic *β* cells but its molecular mechanism will be investigated in the future.

In conclusion, the present study provides evidence for the first time for insulin releasing protein fractions in Korean mistletoe. It is required for separating a large amount of specific single peptide from KME which has an insulin-secreting activity. Together with the current findings, it is thought that this single peptide from KME can represent a source of potential new therapy for plant treatment of diabetes.
